# Causal effects of circulating cathepsins on cholecystitis, cholelithiasis, primary biliary cholangitis, primary sclerosing cholangitis, acute pancreatitis, and chronic pancreatitis: A Mendelian randomization study

**DOI:** 10.1097/MD.0000000000050809

**Published:** 2026-09-18

**Authors:** Tian Zou, Yuehang Fan, Ning Xuan, Xiangyu Meng, Yuji Jiang

**Affiliations:** a Department of Nephrology, Yanbian University Hospital, Yanji, Jilin, China.

**Keywords:** biliary disorders, cathepsins, causality, gallbladder diseases, Mendelian randomization, pancreatic diseases, single nucleotide polymorphisms

## Abstract

Gallbladder, biliary, and pancreatic disorders are nonneoplastic diseases marked by inflammation, epithelial injury, and fibrosis. Cathepsins are lysosomal proteases involved in inflammatory activation, immune regulation, extracellular matrix turnover, and pancreatic enzyme activation. Observational links between dysregulated cathepsins and hepatopancreatobiliary pathology could reflect confounding or reverse causation. This study used Mendelian randomization (MR) to assess whether circulating cathepsins causally influence gallbladder, biliary, and pancreatic diseases. We conducted a 2-sample MR analysis using genetic variants associated with circulating cathepsin levels from the INTERVAL genome-wide association studies (3301 European participants). Summary statistics for cholecystitis, cholelithiasis, primary biliary cholangitis (PBC), primary sclerosing cholangitis, acute pancreatitis, and chronic pancreatitis were extracted from European-ancestry genome-wide association studies consortia. Instrumental variables were selected at *P* < 5 × 10^−6^ with linkage disequilibrium *r*^2^ < 0.001 to maximize instrument availability while retaining independence. The inverse-variance weighted estimator was the primary model, complemented by weighted median, MR-Egger, and mode-based methods. Instrument strength, pleiotropy, and heterogeneity were evaluated via *F* statistics, MR-Egger intercept, MR Pleiotropy RESidual Sum and Outlier, and Cochran *Q*. Benjamini–Hochberg false discovery rate (FDR) correction accounted for multiple testing, and sensitivity analyses included leave-one-out tests and PhenoScanner filtering. Two cathepsin B associations remained significant after FDR correction. Higher genetically predicted cathepsin B was linked to lower PBC risk (odds ratio [OR] = 0.784; 95% confidence interval [CI]: 0.664–0.925; *P* = .004; FDR = 0.022) and higher cholecystitis risk (OR = 1.080; 95% CI: 1.022–1.141; *P* = .006; FDR = 0.018). Other nominal associations did not survive correction and should be interpreted cautiously: cathepsin O with cholelithiasis (OR = 1.001; 95% CI: 1.000–1.003; *P* = .033; FDR = 0.199), cathepsin L2 with acute pancreatitis (OR = 1.156; 95% CI: 1.008–1.327; *P* = .038; FDR = 0.227), and cathepsin H with chronic pancreatitis (OR = 0.922; 95% CI: 0.853–0.997; *P* = .043; FDR = 0.255). No heterogeneity, horizontal pleiotropy, or reverse causation was detected. Genetically predicted cathepsin B appears protective for PBC but increases cholecystitis risk, supporting distinct causal roles among circulating cathepsins in hepatopancreatobiliary diseases. Associations involving cathepsin O, L2, and H were nominal, warranting cautious interpretation, replication, and further functional studies.

## 1. Introduction

Gallbladder and biliary diseases affect a substantial portion of the elderly population and their incidence is rising globally, underscoring the urgent need for elucidating underlying molecular mechanisms in aging digestive pathology.^[1,2]^ The pathogenesis of gallbladder, biliary tract, and pancreatic diseases is multifaceted, involving a variety of risk factors and intricately linked to derangements in proteolytic enzyme activity and cellular protein metabolism.^[3]^ Cathepsins, which act as lysosomal proteases involved in intracellular protein degradation, are increasingly recognized for their roles in inflammation, fibrosis, and tissue remodeling of the digestive system. Elevated cathepsin expression has been observed in pancreatic and biliary tissues of patients with chronic pancreatitis (CP) and cholangiopathies.^[4]^ Human tissues express roughly 15 cathepsin enzymes (Z, W, S, O, L2, L, K, H, G, F, E, D, C, B, and A), and multiple members of this family have been implicated as biomarkers or risk determinants in diverse pathological conditions.^[5]^

Cathepsins are lysosomal proteases that extend beyond bulk protein degradation: in inflammatory tissues they support antigen processing in endolysosomal compartments and thereby shape immune-mediated tissue injury.^[6]^ Dysregulated cathepsin activity can also promote extracellular matrix (ECM) remodeling and extracellular proteolysis, processes relevant to tissue remodeling and fibrosis across hepatopancreatobiliary disorders.^[7]^ Consistent with this, cathepsin B can modulate inflammatory signaling by promoting NOD-like receptor pyrin domain containing 3 inflammasome activation and downstream pro-inflammatory cytokine release, and it also contributes to immune regulation, apoptosis, and ECM degradation, thereby potentially explaining its observed effects on hepatopancreatobiliary disease risk.^[8]^ For cholelithiasis, cathepsin O, as a cysteine cathepsin, is plausibly involved in stone-relevant pathophysiology by contributing to ECM remodeling and extracellular proteolysis that can alter the gallbladder microenvironment and promote cholesterol crystallization.^[9]^ For acute pancreatitis (AP), cathepsin L2 (cathepsin V) is a lysosomal cysteine protease functionally homologous to cathepsin L, and cathepsin L-dependent regulation of trypsinogen/trypsin activity supports its mechanistic plausibility in pancreatitis development.^[10]^ Finally, cathepsin H is expressed in the pancreas and participates in lysosomal protease biology and protease homeostasis, suggesting a potential protective role in CP progression.^[11]^

Mendelian randomization (MR) leverages genetic variants as instrumental variables (IVs) to infer causal relationships between circulating exposures and disease outcomes, reducing confounding and reverse causation inherent to observational studies.^[12–15]^ Given the growing availability of large-scale proteomic and disease genome-wide association studies (GWAS) data, MR provides an efficient and ethically feasible framework for evaluating whether genetically proxied cathepsin levels influence hepatopancreatobiliary disease risk. Prior MR work has suggested causal links between cathepsin B and biliary tract cancer,^[16]^ as well as connections between cathepsins and inflammatory bowel disease,^[17]^ supporting the utility of this approach in protease-related etiologic research.

Building on these precedents, we hypothesized that genetically predicted circulating cathepsin levels exert causal effects on gallbladder, biliary, and pancreatic disease susceptibility, potentially in a cathepsin-specific manner. The primary objectives of this study were to systematically assess the causal relationships between circulating levels of multiple cathepsins and risks of cholecystitis, cholelithiasis, primary biliary cholangitis (PBC), primary sclerosing cholangitis (PSC), AP, and CP, using 2-sample MR while carefully addressing instrument strength, pleiotropy, and multiple testing correction.

## 2. Materials and methods

### 2.1. MR data and process

**Figure 1.**
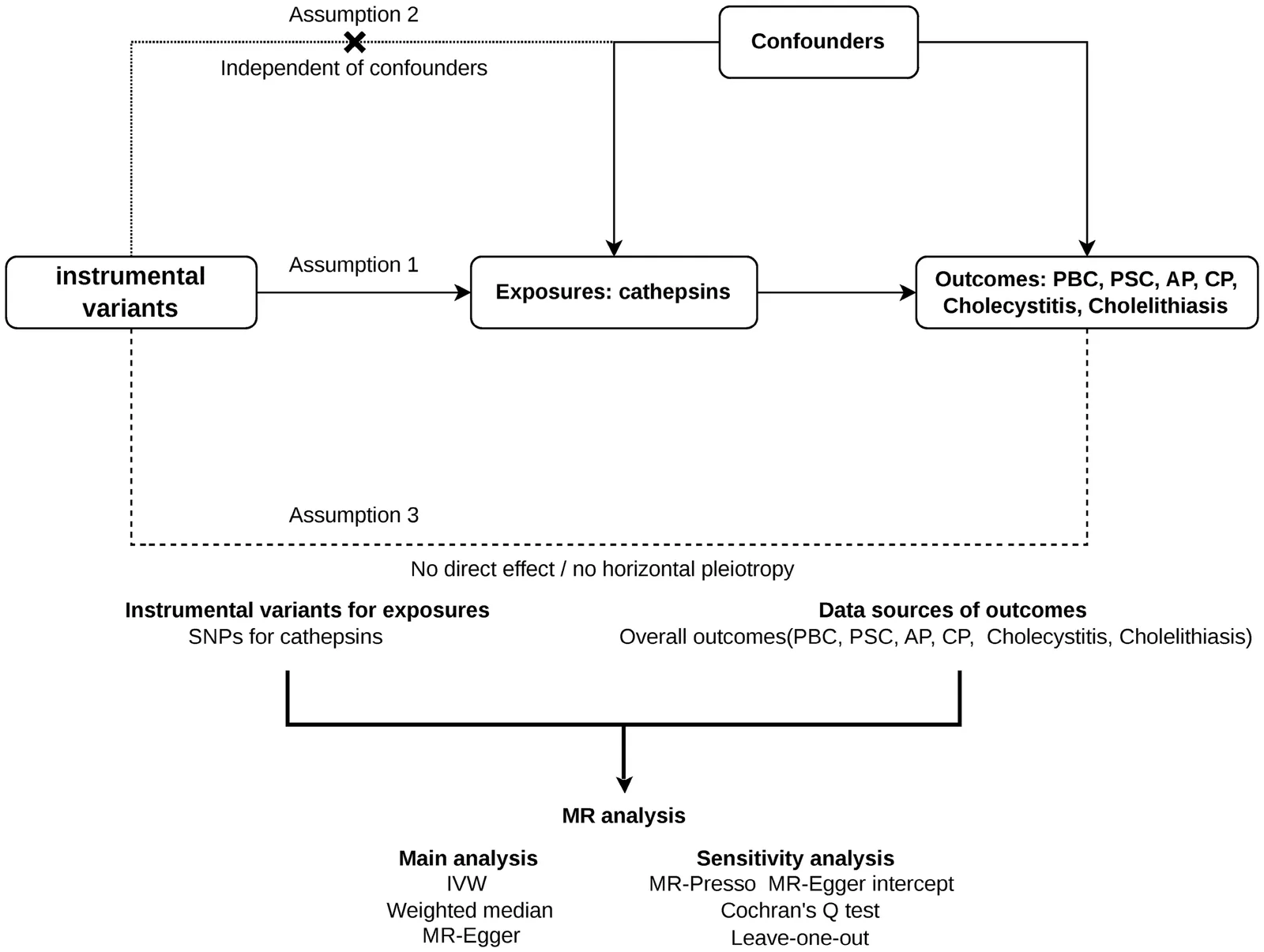
Flowchart of the MR analysis.This investigation adhered to the 3 core assumptions underlying Mendelian randomization. Assumption 1: As illustrated by the solid arrow, the chosen instrumental variables (IVs) must exhibit a robust association with the exposure, namely the circulating levels of cathepsins. Assumption 2: As depicted by the dashed lines, the instrumental variables are assumed to be independent of any confounders that could simultaneously affect the exposure and the outcomes. Assumption 3: The IVs influence the outcomes exclusively through the exposure, without affecting the outcome via alternative causal pathways.AP = acute pancreatitis; CP = chronic pancreatitis, IVW = inverse-variance weighted, MR = Mendelian randomization, PBC = primary biliary cholangitis, PSC = primary sclerosing cholangitis, SNP = single nucleotide polymorphism.

A 2-sample MR framework was utilized to examine whether circulating cathepsin levels exert causal influences on diseases of the gallbladder, biliary tract, and pancreas. The overall study design and analytical workflow are illustrated in Figure 1.

### 2.2. IVs selection

The single nucleotide polymorphisms (SNPs) selected for this study were required to satisfy the 3 fundamental assumptions of MR: relevance assumption: the validity of an instrument requires that it be strongly linked to the exposure of interest, here represented by circulating cathepsin concentrations. Independence assumption: the SNPs used as instruments should be free from correlations with any confounding variables, recognized or unrecognized, that could impact both the exposure and the outcomes. Exclusion restriction assumption: the SNPs should affect the risk of gallbladder, biliary, and pancreatic diseases exclusively through their association with cathepsin levels, without influencing the outcomes via alternative biological pathways.

To ensure that the selected variants satisfied the MR assumptions, we applied strict inclusion criteria: genetic variants linked to circulating cathepsin concentrations were identified using a GWS criterion of *P* < 5 × 10^−6^. Rather than using the conventional genome-wide significance threshold (*P* < 5 × 10^−8^), we adopted a relaxed genome-wide suggestive threshold (*P* < 5 × 10^−6^) to retain an adequate number of instruments for proteomic/omics exposures, while still limiting false positives. To mitigate potential weak-instrument bias, instrument strength was evaluated using *F* statistics; we required *F* > 10 for all cathepsins (Table S1, Supplemental Digital Content 1). Furthermore, all included SNPs were required to be independent of 1 another, with linkage disequilibrium (LD) controlled at *r*^2^ < 0.001 and a physical distance of at least 10,000 kb between any pair of variants.

The strength of the IVs was evaluated using the *F* statistic to assess the relevance assumption of MR. The strength of the instruments was assessed by calculating the *F*-statistic as

*F* = *R*^2^ × (N − *k* − 1)/ [(1 − *R*^2^) × k]. In this expression, N refers to the GWAS sample size for circulating cathepsins, *k* corresponds to the number of SNP instruments, and *R*^2^ reflects the variance in cathepsin concentrations explained by each variant. The value of *R*^2^ was derived using the formula: *R*^2^ = 2 × β^2^ × EAF × (1 − EAF), where β represents the estimated genetic effect of each SNP on cathepsin levels, and EAF is the effect allele frequency. An *F* statistic >10 was considered indicative of sufficient instrument strength, suggesting a strong association between the selected SNPs and the exposure.

### 2.3. SNPs associated with cathepsins

Genetic variants associated with circulating cathepsin levels were derived from the INTERVAL study, which comprised 3301 participants of European ancestry. Written informed consent was obtained from all individuals, and ethical approval for the study was granted by the National Research Ethics Service (approval number: 11/EE/0538). The corresponding summary-level GWAS data are accessible through the Integrative Epidemiology Unit OpenGWAS database. All cathepsin-associated SNPs included in this analysis satisfied the genome-wide suggestive significance threshold (*P* < 5 × 10^−6^) and were confirmed to be independent of each other, with no LD detected (*r*^2^ < 0.001, distance ≥ 10,000 kb). Comprehensive information regarding the selected IVs is provided in Table S1, Supplemental Digital Content 1. All selected instruments were screened in PhenoScanner. SNPs displaying genome-wide associations with liver/biliary/pancreatic traits or autoimmune phenotypes were excluded to mitigate horizontal pleiotropy. The full list of excluded variants and their mapped traits is provided in Table S3, Supplemental Digital Content 2.

### 2.4. SNPs associated with gallbladder, biliary, and pancreatic diseases

Our study encompassed 6 nonneoplastic digestive diseases, including cholecystitis, cholelithiasis, PBC, PSC, AP, and CP. Detailed summary statistics for these outcome datasets are provided in Table 1.

**Table 1:** Basic characteristics of the GWAS datasets included in this analysis.

Outcomes	Consortium	Sample size (cases/controls)	Population
Cholecystitis	Sakaue S et al.	9820/461,431	European
Cholelithiasis	Dönertaş HM et al.	7895/476,703	European
Primary biliary cholangitis	Cordell HJ et al.	2764/10,475	European
Primary sclerosing cholangitis	Ji SG et al.	2871/12,019	European
Acute pancreatitis	FinnGen	3022/195,144	European
Chronic pancreatitis	FinnGen	1737/195,144	European

GWAS = genome-wide association studies.

For the outcome datasets, GWAS summary statistics for 6 digestive system diseases were used. SNPs associated with cholecystitis were obtained from a GWAS by Sakaue S et al,^[18]^ which included 9820 cases and 461,431 controls of European ancestry. Data for cholelithiasis were derived from Dönertaş HM et al,^[19]^ comprising 7895 cases and 476,703 controls. Summary statistics for PBC were sourced from Cordell HJ et al,^[20]^ including 2764 cases and 10,475 controls. For PSC, GWAS data from Ji SG et al^[21]^ were used, involving 2871 cases and 12,019 controls. The datasets for AP and CP were obtained from publicly available European GWAS summary data, including 3022 cases with 195,144 controls and 1737 cases with 195,144 controls, respectively.

Disease phenotypes were defined according to the phenotype definitions used in the original GWAS datasets. For UK Biobank-based ebi-a datasets, algorithmically defined outcomes were generated by combining self-reported information with hospital inpatient diagnosis records and death registry data. Specifically, cholecystitis cases were identified using International Classification of Diseases, 10th revision (ICD-10) code K81 (cholecystitis), and cholelithiasis cases were identified using ICD-10 code K80 (cholelithiasis). For PBC, cases included in the Cordell et al dataset fulfilled the American Association for the Study of Liver Diseases criteria for PBC. For PSC, a diagnosis of PSC was based on standard clinical, biochemical, cholangiographic and histological criteria, with exclusion of secondary causes of sclerosing cholangitis. For FinnGen-derived pancreatitis endpoints, case definitions were based on registry-based ICD-10 codes: AP was defined using hospital discharge ICD-10 K85 and cause-of-death ICD-10 K85, and CP was defined using hospital discharge ICD-10 K86.00, K86.01, K86.08, and K86.1 together with corresponding ICD-10 codes in the cause-of-death registry. All included participants were of European descent, and detailed dataset information is summarized in Table S2, Supplemental Digital Content 3.

The exposure GWAS (INTERVAL) and all outcome consortia represent distinct study populations. INTERVAL comprises 3301 blood donors recruited for proteomic profiling, whereas the outcome datasets were generated by separate large-scale consortia (Sakaue et al., Dönertaş et al., Cordell et al., Ji et al., FinnGen) based on independent European cohorts such as UK Biobank hospital records, Finnish biobank registries, and disease-specific consortia. No participant overlap between INTERVAL and these outcome GWAS is reported in the original publications, and the datasets draw from geographically and operationally separate sources. Therefore, sample overlap is unlikely to affect our 2-sample MR inference. Although we cannot guarantee zero overlap (since individual-level data are not available), the nonoverlapping recruitment frames and the lack of reported shared participants suggest that any overlap, if present, would be minimal and unlikely to materially bias the results.

### 2.5. MR analysis

To explore whether circulating cathepsin levels exert causal influences on diseases of the gallbladder, biliary tract, and pancreas, 5 complementary MR methods were implemented, including the inverse-variance weighted (IVW) model, weighted median estimator, MR-Egger regression, simple mode, and weighted mode. The IVW method was used as the primary analytical approach, as it provides an efficient pooled estimate by combining the effects of individual SNPs through a weighted regression model.

This approach yields unbiased causal estimates when all IVs are valid and there is no directional pleiotropy among SNPs.^[22]^ In addition, the weighted median approach was utilized, as it generates reliable causal estimates under the condition that no less than half of the cumulative instrument weight is contributed by valid variants, even if up to 50% of the instruments violate MR assumptions.^[23]^ The MR-Egger approach was utilized to detect and rectify horizontal pleiotropy, which is defined as genetic variants influencing the outcomes via mechanisms that are independent of circulating cathepsin levels.^[24]^

All analyses were implemented in R (maintained by The R Foundation for Statistical Computing) using the TwoSampleMR toolkit (developed by the MRC Integrative Epidemiology Unit at the University of Bristol). Causal effects are presented as odds ratios (ORs) accompanied by corresponding 95% confidence intervals (CIs). An OR >1 indicated that circulating cathepsin levels increased the risk of the respective outcome, whereas an OR <1 suggested a protective association. The instrument counts and results are summarized in Table S4, Supplemental Digital Content 4. Because we evaluated 54 exposure–outcome pairs, IVW *P* values were corrected for multiple testing via the Benjamini–Hochberg false discovery rate (FDR) procedure; associations with FDR < 0.05 were considered robust, while nominal findings (uncorrected *P* < .05 but FDR > 0.05) were interpreted with caution. Statistical power for each primary IVW analysis was estimated using the mRnd tool (an open-source online application developed by Centre for Neurogenetics and Statistical Genomics [CNS Genomics], https://shiny.cnsgenomics.com/mRnd/; Table 2).

**Table 2:** Statistical power for the primary IVW estimates.

Exposure	Outcome	Method	Power
Cathepsin B	Primary biliary cholangitis	IVW	0.98
Cathepsin B	Cholecystitis	IVW	0.95
Cathepsin O	Cholelithiasis	IVW	0.05
Cathepsin L2	Acute pancreatitis	IVW	0.72
Cathepsin H	Chronic pancreatitis	IVW	0.49

IVW = inverse-variance weighted.

### 2.6. Sensitivity analysis

The intercept term from the MR-Egger regression was evaluated to determine whether horizontal pleiotropy was present. A nonsignificant intercept (*P* > .05) was considered to indicate the absence of directional pleiotropy. Furthermore, horizontal pleiotropy was evaluated using the MR Pleiotropy RESidual Sum and Outlier (MR-PRESSO) method, which functions by detecting and excluding outlier genetic instruments that may distort the causal inference.^[25]^

To evaluate the heterogeneity among the IVs, Cochran *Q* test was carried out. A *P* value exceeding .05 suggests the absence of evidence for heterogeneity among the SNP-specific estimates. To investigate whether any individual instrumental variant exerted a disproportionate influence on the causal estimates, a leave-one-out analysis was implemented. Each SNP was removed in turn, and the MR analysis was repeated to evaluate changes in effect size. By applying this procedure, influential variants can be detected, enabling an assessment of the stability of the causal inferences and enhancing the clarity and robustness of the results.

### 2.7. Reverse MR analysis

To assess the possibility of reverse causation, we performed reverse MR analyses in which disease phenotypes were treated as the exposures and circulating cathepsin levels were treated as the outcomes. Instrument selection for the reverse direction followed the same criteria as the primary analyses (*P* < 5 × 10^−6^, LD *r*^2^ < 0.001, and physical distance ≥ 10,000 kb). After harmonization of effect alleles between exposure and outcome datasets, causal effects were estimated using IVW as the primary method. The instrument counts and results are summarized in Table S5, Supplemental Digital Content 5.

### 2.8. Ethical review

This study used publicly available aggregate statistical data. Ethical approval and informed consent had been obtained in the original studies. No additional ethical approval was required for the present study.

## 3. Results

### 3.1. Findings from the 2-sample MR analysis

**Figure 2.**
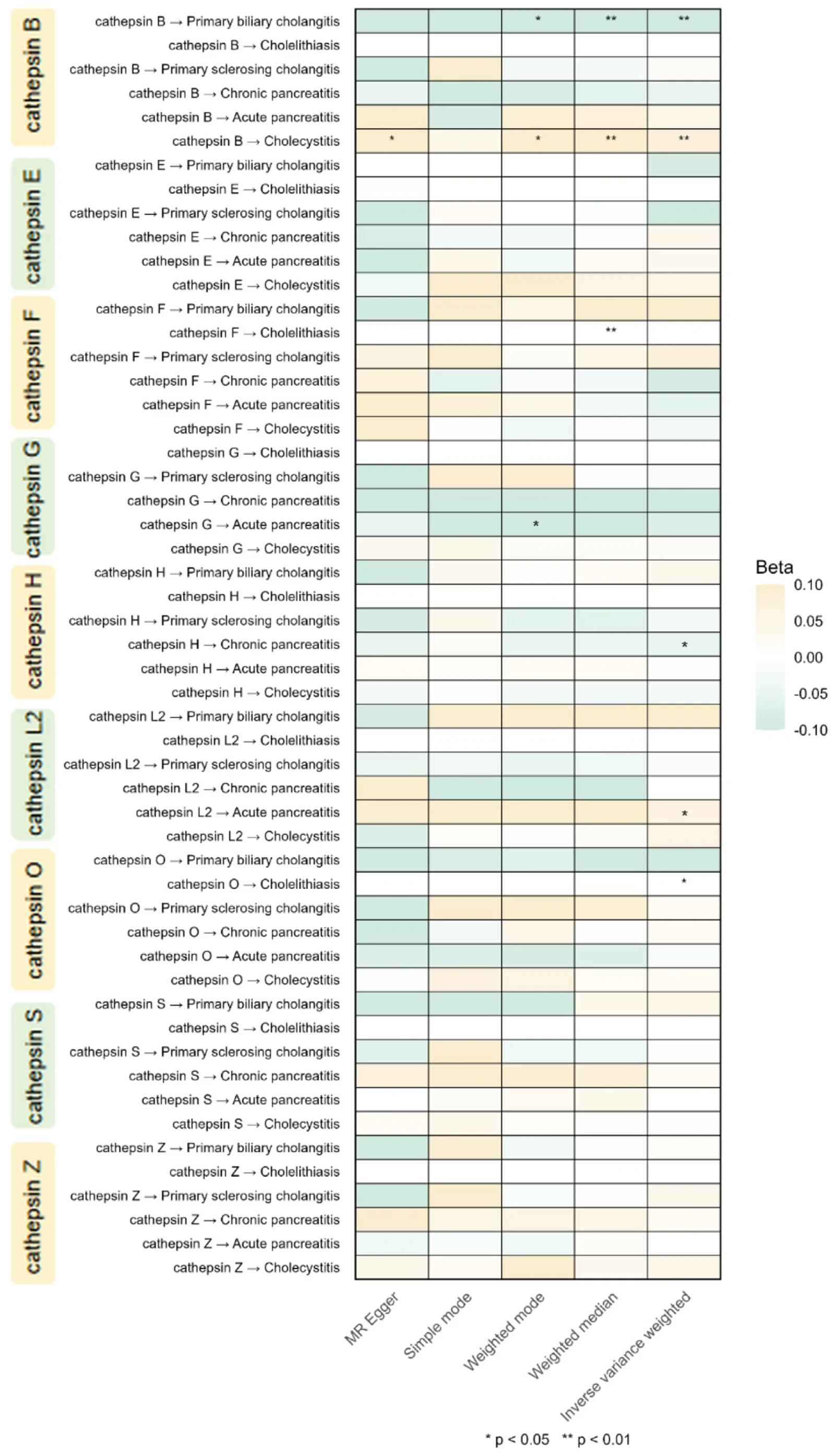
Heatmap summarizing estimated causal effects of genetically predicted circulating cathepsin levels on 6 digestive system disorders across 5 Mendelian randomization (MR) methods. Each cell displays the beta coefficient (effect direction and magnitude) for a given cathepsin–outcome pair and analysis method. Color indicates the direction of the estimated effect: yellow corresponds to positive associations and green corresponds to negative associations; darker shading reflects larger absolute effect magnitude. Statistical significance is indicated by asterisks (* *P* < .05, ** *P* < .01).

**Figure 3.**
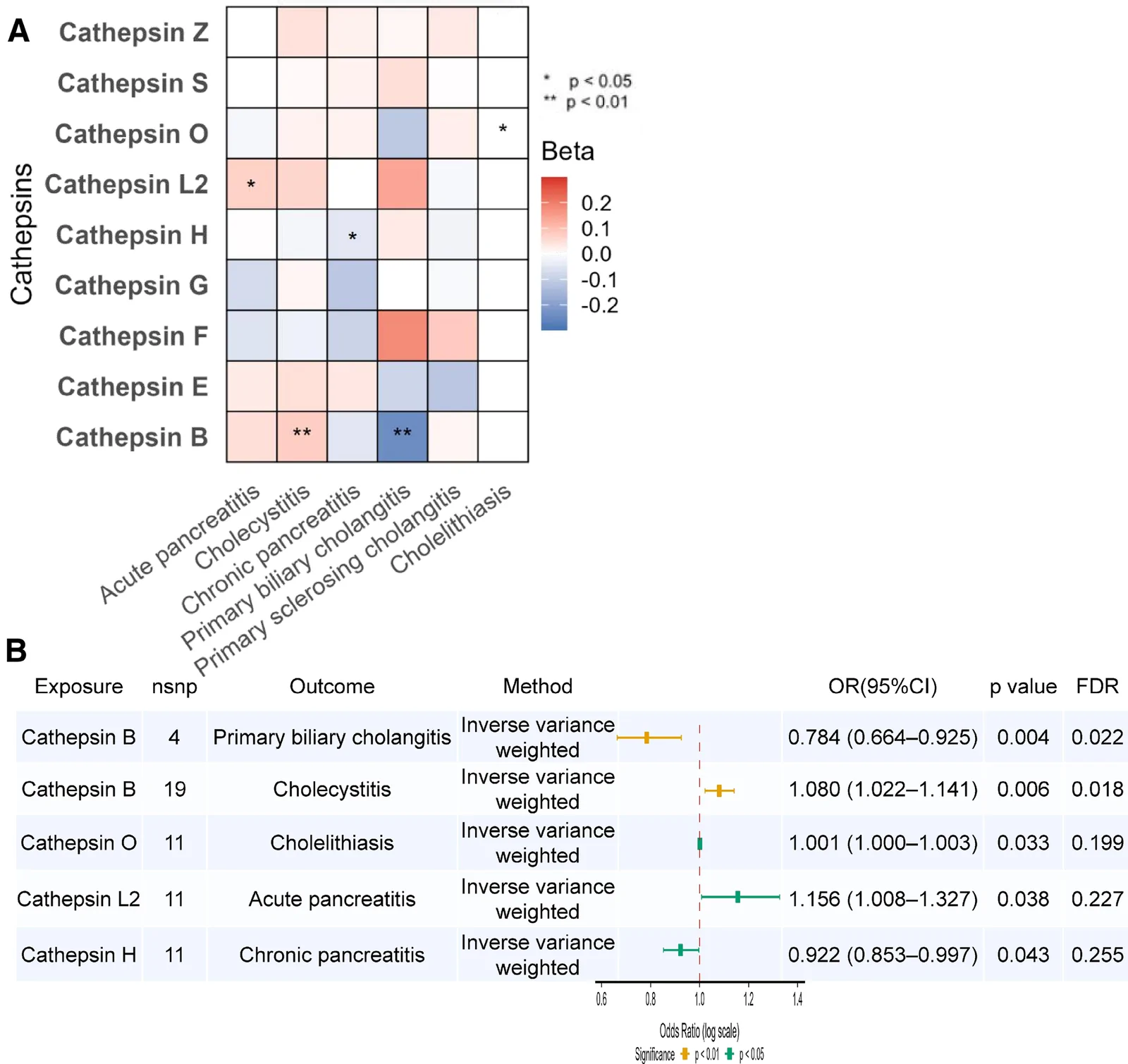
MR analysis reveals the causal associations between circulating cathepsins and 6 digestive system diseases. (A) Heatmap generated from the IVW analysis, illustrating the causal relationships of cathepsin B, cathepsin L2, cathepsin H, and cathepsin O with disorders of the gallbladder, biliary tract, and pancreas. (B) Forest plot illustrating the causal estimates of these significant associations identified through the IVW analysis. CI = confidence interval, FDR = false discovery rate, IVW = inverse-variance weighted, MR = Mendelian randomization, OR = odds ratio.

Our study examined 9 subtypes of cathepsins, namely B, E, F, G, H, L2, O, S, and Z, and performed MR analyses to explore their potential causal relationships with 6 digestive system diseases, including gallbladder, biliary, and pancreatic disorders. The associations and effect estimates derived from multiple MR methods are summarized in Figure 2. A heatmap illustrating the causal estimates (β values) of circulating cathepsins on 6 digestive system diseases obtained from IVW methods is presented in Figure 3A. The analysis revealed that higher genetically predicted cathepsin B was associated with lower PBC risk (OR = 0.784; 95% CI: 0.664–0.925; *P* = .004; FDR = 0.022) and increased cholecystitis risk (OR = 1.080; 95% CI: 1.022–1.141; *P* = .006; FDR = 0.018), both of which remained significant after FDR correction. Cathepsin O showed a nominally positive association with cholelithiasis (OR = 1.001; 95% CI: 1.000–1.003; *P* = .033; FDR = 0.199) that is very close to the null value and did not survive multiple-testing adjustment. Cathepsin L2 and cathepsin H produced nominal associations with acute (OR = 1.156; 95% CI: 1.008–1.327; *P* = .038; FDR = 0.227) and CP (OR = 0.992; 95% CI: 0.853–0.997; *P* = .043; FDR = 0.255), respectively, which should be interpreted cautiously given their limited statistical robustness, as detailed in Figure 3B. No statistically significant causal associations were observed for the remaining cathepsin subtypes with respect to the studied disease outcomes (Table S4, Supplemental Digital Content 4). Statistical power for the primary IVW estimates was calculated using the mRnd tool. Table 2 summarizes the power for the key associations.

**Figure 4.**
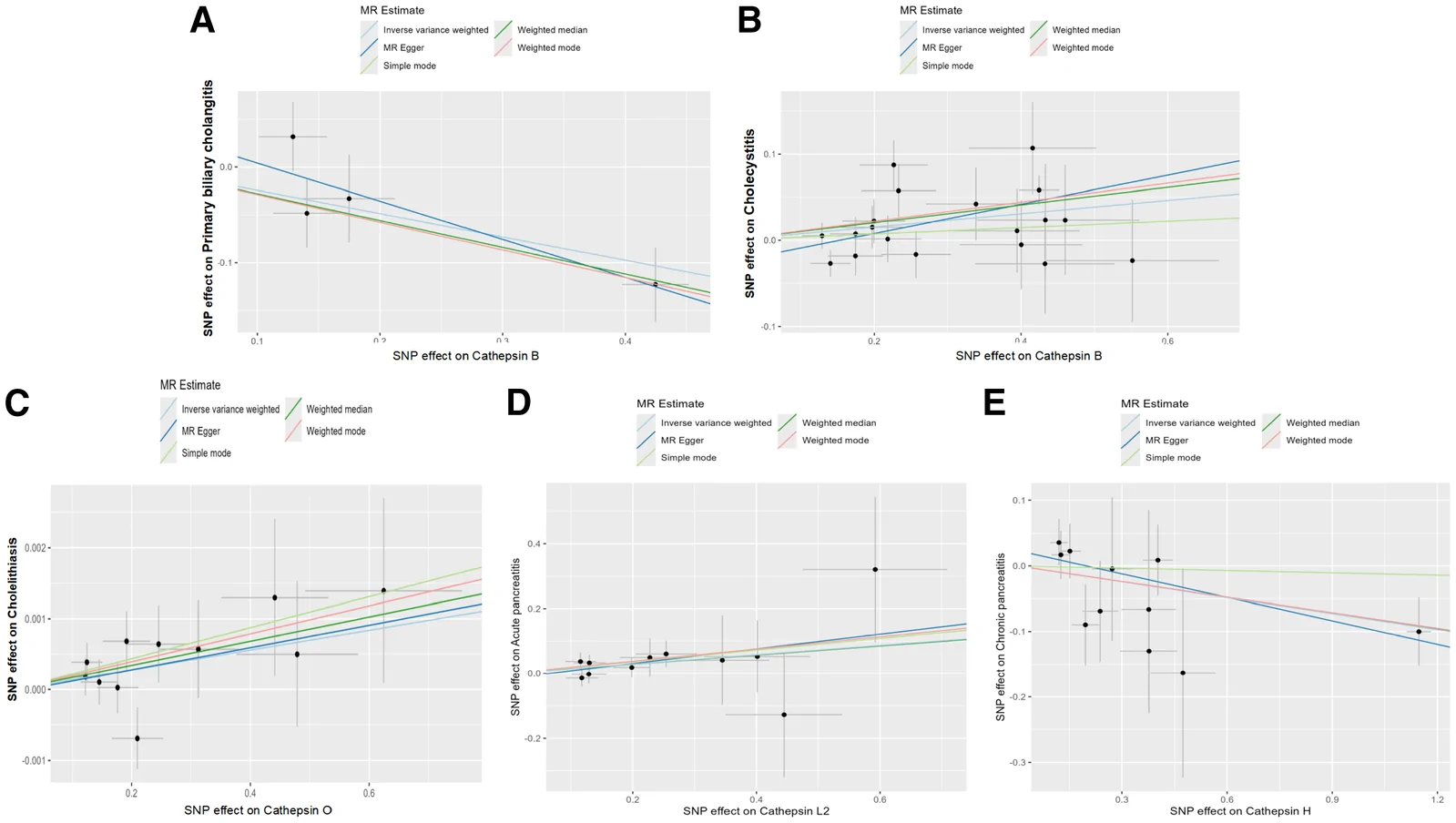
Scatter plots illustrating the sensitivity analyses: (A) cathepsin B with primary biliary cholangitis; (B) cathepsin B with cholecystitis; (C) cathepsin O with cholelithiasis; (D) cathepsin L2 with acute pancreatitis; and (E) cathepsin H with chronic pancreatitis. Lines represent IVW, MR-Egger, weighted median, simple mode, and weighted mode estimates. Corresponding β (or OR), 95% CI, and *P* values are presented in Table S4, Supplemental Digital Content 4, while the number of SNPs and instrument *F* statistics are summarized in Table S1, Supplemental Digital Content 1. MR-Egger intercept, MR-PRESSO global test, and Cochran *Q* statistics are reported in Table 3; no analysis yielded significant pleiotropy (all MR-Egger intercept *P* > .05 and MR-PRESSO global *P* > .05). CI = confidence interval, IVW = inverse-variance weighted, MR = Mendelian randomization, MR-PRESSO = Mendelian randomization Pleiotropy RESidual Sum and Outlier, OR = odds ratio, SNPs = single nucleotide polymorphisms.

**Figure 5.**
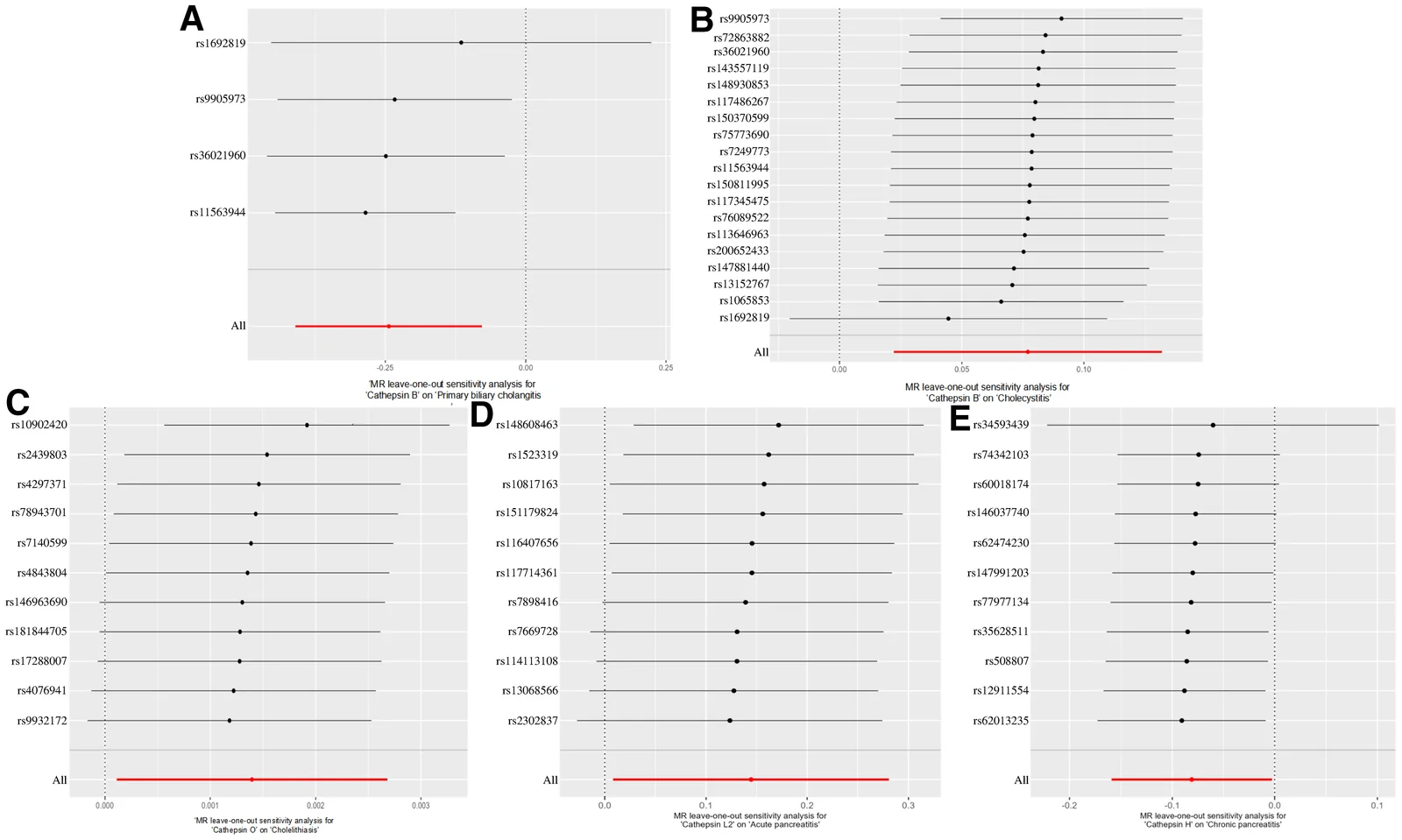
Leave-one-out analysis results: (A) cathepsin B with primary biliary cholangitis; (B) cathepsin B with cholecystitis; (C) cathepsin O with cholelithiasis; (D) cathepsin L2 with acute pancreatitis; and (E) cathepsin H with chronic pancreatitis. Leave-one-out analysis indicates that no single SNP disproportionately influenced the overall MR results, supporting the stability and reliability of the findings. MR = Mendelian randomization, SNP = single nucleotide polymorphism.

Sensitivity analyses revealed robust instrument performance, with the MR-PRESSO procedure identifying no outlier variants. Evaluation of horizontal pleiotropy using MR-Egger and MR-PRESSO tests showed no indication of directional pleiotropy (both *P* > .05), as presented in Figure 4. Additionally, Cochran *Q* test suggested an absence of significant heterogeneity across instruments (*P* > .05). Comprehensive results from the sensitivity analyses appear in Table 3. Furthermore, the leave-one-out analysis confirmed that removal of individual SNPs did not meaningfully alter the causal estimates, underscoring the stability and internal consistency of the MR findings (Fig. 5).

**Table 3:** Findings from sensitivity and heterogeneity analyses.

Exposures	Outcomes	Cochran *Q* statistic	*P* value for Cochran *Q*	*P* value for intercept	MR-PRESSO global test
Cathepsin B	Cholecystitis	21.571	.202	.121	0.133
Cathepsin B	Primary biliary cholangitis	2.214	.331	.397	0.446
Cathepsin O	Cholelithiasis	8.075	.527	.901	0.720
Cathepsin L2	Acute pancreatitis	5.130	.823	.652	0.890
Cathepsin H	Chronic pancreatitis	6.077	.732	.336	0.809

MR-PRESSO = Mendelian randomization Pleiotropy RESidual Sum and Outlier.

### 3.2. Reverse MR analysis

**Figure 6.**
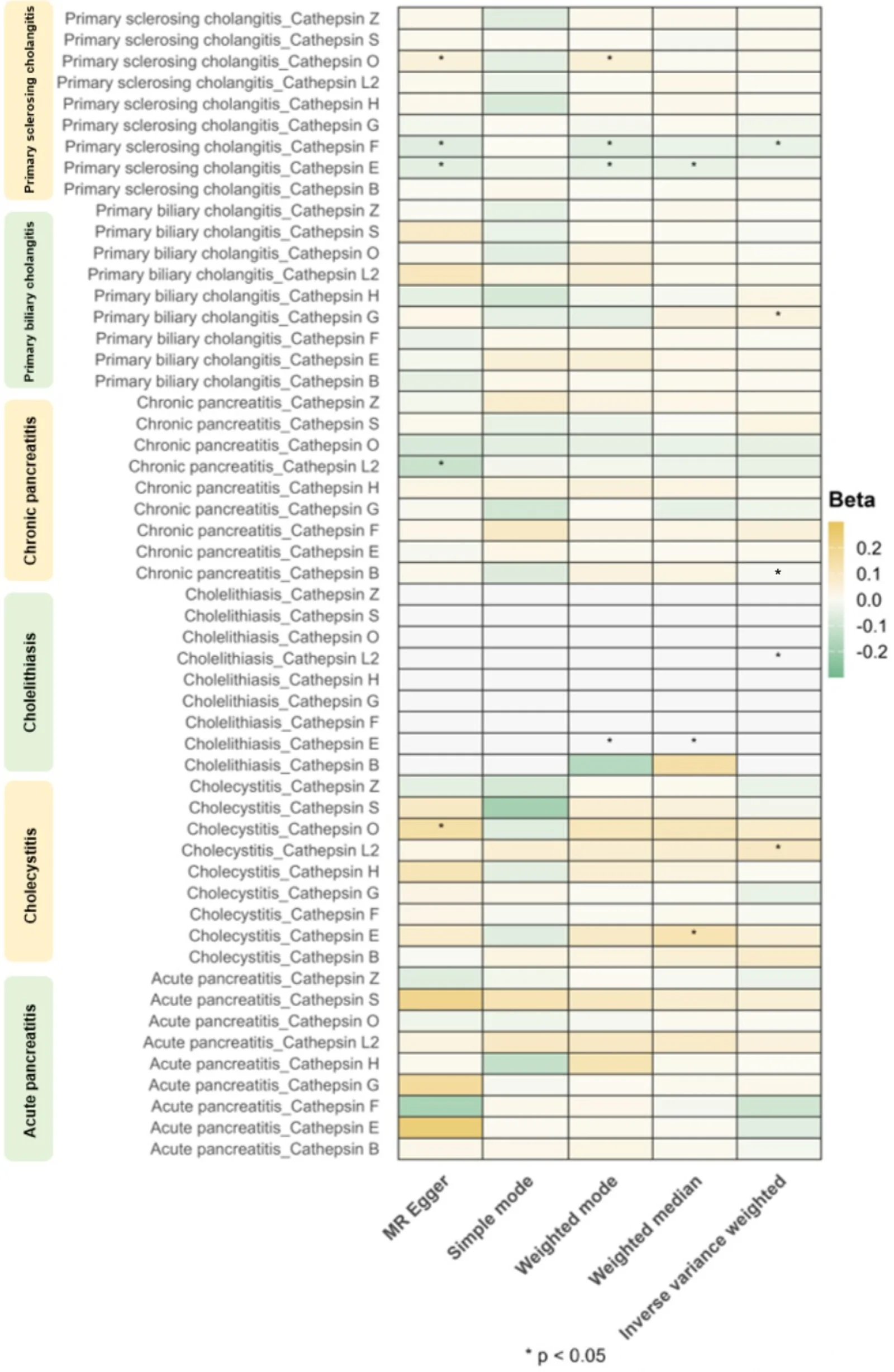
Heat map of reverse Mendelian randomization analyses showing beta estimates for 6 digestive system diseases against 9 circulating cathepsins, with particular emphasis on the IVW results. Each row represents 1 disease–cathepsin pair, and each column depicts a different MR estimator; colors indicate the direction and magnitude of the beta estimates, and asterisks denote nominal significance (*P < .05). While the figure presents every disease–cathepsin combination for completeness, our interpretation focuses on the 5 exposure–outcome pairs that showed notable forward MR associations (cathepsin B → PBC/cholecystitis, cathepsin O → cholelithiasis, cathepsin L2 → acute pancreatitis, and cathepsin H → chronic pancreatitis). The IVW estimates for these 5 pairs do not provide consistent evidence of reverse causality on circulating cathepsin levels. IVW = inverse-variance weighted, MR = Mendelian randomization, PBC = primary biliary cholangitis.

Reverse MR assessment explored the possibility that 6 digestive diseases may causally affect circulating cathepsins. According to the IVW estimates, no causal relationships were identified for cathepsin B with PBC or cholecystitis, cathepsin O with cholelithiasis, cathepsin L2 with AP, or cathepsin H with CP. A detailed summary of the MR findings is provided in Figure 6 (Table S5, Supplemental Digital Content 5).

## 4. Discussion

The onset and progression of gallbladder, biliary tract, and pancreatic diseases are a multifactorial process influenced by age, sex, genetic predisposition, and metabolic conditions such as obesity and dyslipidemia, with several pathways linked to abnormal protein and enzyme metabolism.^[26]^ Cathepsins, a family of lysosomal cysteine proteases, regulate both intracellular protein degradation and ECM remodeling. Aberrant activation of cathepsins has been implicated in the progression of gallbladder, biliary, and pancreatic diseases through promoting inflammation, fibrosis, and cell death. Aberrant cathepsin activity has been reported in hepatobiliary and pancreatic disease states and is associated with inflammation, fibrosis, and tissue injury.^[27–29]^ However, such observations may represent downstream responses to established disease processes and do not necessarily imply that cathepsins initiate disease. In contrast, MR tests whether genetically predicted circulating cathepsin levels are likely to causally influence disease susceptibility. Therefore, we adopted an MR framework to evaluate the causal effects of circulating cathepsins on gallbladder, biliary, and pancreatic disorders from a genetic perspective.

Cathepsins are a group of proteases involved in diverse inflammatory and immune-mediated conditions of the digestive system, such as liver fibrosis and inflammatory bowel disease.^[30]^ To date, no studies have evaluated whether circulating cathepsins causally influence gallbladder, biliary, or pancreatic disorders. To our knowledge, no previous MR study has evaluated the causal effects of circulating cathepsins on nonneoplastic gallbladder, biliary, and pancreatic diseases. Cathepsins comprise a family of lysosomal proteases (including cysteine, serine, and aspartic members) that generally participate in intracellular protein turnover, antigen processing, and tissue remodeling.^[31]^ Under physiological conditions, they are sequestered within acidic compartments, but pathological stressors can promote lysosomal leakage, releasing these proteases into the cytoplasm where they may influence apoptotic and inflammatory cascades.^[32–34]^ While dysregulated cathepsin activity has been observed in cholestatic and inflammatory conditions, our MR analysis specifically interrogates whether genetically predicted circulating levels – even before overt disease – causally influence risk. The genetic findings suggest that cathepsin B has bidirectional roles (protective for PBC but detrimental for cholecystitis), cathepsin O and L2 are nominally associated with cholelithiasis and AP, respectively, and cathepsin H appears protective against CP.

Cathepsin B is a lysosomal cysteine protease of the papain family with high expression in hepatic and pancreatic tissue, as well as in innate immune cells such as macrophages. Although it displays maximal activity in acidic lysosomal compartments, it can retain limited catalytic function at neutral pH and has been implicated in intracellular protein turnover, autophagy, and immune responses. Observational and experimental studies have linked aberrant Cathepsin B expression to inflammatory signaling (e.g., nuclear factor kappa-light-chain-enhancer of activated B cells/NOD-like receptor pyrin domain containing 3 activation), apoptotic pathways, and ECM remodeling in hepatobiliary tissues,^[35–37]^ but these findings primarily reflect disease-associated changes rather than causal roles. Our MR results indicate that genetically predicted higher Cathepsin B levels are associated with reduced risk of PBC yet increased risk of cholecystitis. This bidirectional pattern suggests that cathepsin B may influence biliary homeostasis through context-dependent modulation of inflammation or immune clearance, but the specific pathways remain to be elucidated experimentally. Importantly, the MR design supports a potential upstream contribution of cathepsin B to disease susceptibility, rather than simply reflecting downstream upregulation during tissue injury.

Cathepsin O is a cysteine-type lysosomal protease whose activity peaks under acidic conditions, but compared with other family members such as cathepsins B, K, and L, it has been less intensively studied. Emerging observational data have linked cathepsin O to macrophage lipid handling and ECM remodeling in inflammatory settings,^[38]^ yet these associations do not establish causality. To our knowledge, the present study is the 1st MR investigation assessing whether genetically predicted circulating cathepsin O influences cholelithiasis risk. We detected a nominally positive association (OR = 1.001; *P* = .033; FDR = 0.199), but the effect size is extremely close to the null and the analysis had only 5% power, so this finding should be interpreted with considerable caution. While it remains plausible that cathepsin O could affect gallstone pathogenesis through macrophage-mediated ECM turnover or alterations in gallbladder wall integrity, mechanistic validation in experimental models is needed before attributing a causal role.^[39]^

Cathepsin L2 (also known as cathepsin V) is a lysosomal cysteine protease that is highly expressed in epithelial and immune-related tissues, including the liver and pancreas, and exhibits maximal catalytic activity in acidic compartments.^[40]^ Experimental work on cysteine cathepsins more broadly has implicated lysosomal destabilization and cathepsin-mediated trypsinogen activation in the early phases of AP, suggesting these enzymes can influence acinar injury and inflammatory signaling.^[41]^ Given the sequence and functional similarity between cathepsin L and L2, it is plausible that cathepsin L2 participates in similar pathways, although direct evidence remains limited. Our MR analysis provided a nominally positive association between genetically predicted cathepsin L2 and AP (OR = 1.156; *P* = .038; FDR = 0.227; power = 0.72). While this finding is consistent with the broader role of cysteine cathepsins in pancreatic inflammation, the effect is modest and did not survive multiple testing correction; therefore, additional studies, ideally experimental validation or larger genetic datasets, are needed to clarify whether cathepsin L2 plays a causal role in initiating pancreatic injury versus being upregulated secondary to inflammation.^[42,43]^

Cathepsin H is a lysosomal cysteine protease with both endopeptidase and aminopeptidase activities that operates optimally in acidic compartments such as lysosomes and late endosomes.^[44]^ Compared with other cathepsins, it has been less intensively studied, but prior reports have implicated cathepsin H in tissue remodeling and immune regulation.^[45]^ In pancreatic contexts, limited data suggest it might help maintain protease homeostasis, possibly by promoting the degradation of prematurely activated enzymes; however, direct mechanistic evidence remains sparse. Our MR analysis represents, to our knowledge, the 1st genetic investigation of whether circulating cathepsin H influences CP risk. The results indicate a nominally protective association (OR = 0.922; *P* = .043; FDR = 0.255; power = 0.49) that did not survive multiple-testing correction, implying the signal may reflect either a true modest causal effect or residual confounding/low power. Further work, both functional experiments and larger genetic studies, is needed to determine whether cathepsin H genuinely contributes to CP pathogenesis or is upregulated secondary to ongoing injury.

This study has several limitations. The exposure GWAS comprised only 3300 participants, necessitating the use of suggestive-significance instruments (*P* < 5 × 10^−6^). Although we enforced stringent clumping and *F* statistic thresholds, such variants may still suffer from weak-instrument bias or inflation of SNP–exposure estimates. Residual horizontal pleiotropy cannot be fully excluded despite PhenoScanner filtering and MR-PRESSO, and summary-level data preclude exploration of nonlinear effects or effect modification. Finally, all datasets are from European-ancestry populations, limiting generalizability.

In summary, our MR analysis provides genetic evidence that circulating cathepsins may play differential roles in hepatopancreatobiliary disorders. Genetically predicted cathepsin B was associated with a decreased risk of PBC but increased cholecystitis susceptibility, while nominal associations were observed between cathepsin O and cholelithiasis, cathepsin L2 and AP, and cathepsin H and CP. These findings are consistent with potential causal effects but do not constitute definitive proof, because MR relies on strong instrument validity and does not exclude residual pleiotropy or other biases. Several of the observed associations are modest in magnitude, have limited statistical power, and did not survive multiple-testing correction; hence, replication in larger datasets and functional follow-up are required before firm biological or clinical inferences can be drawn.

## Acknowledgments

We thank the FinnGen consortium and the IEU OpenGWAS project for providing publicly available GWAS summary statistics used in this study.

## Author contributions

**Conceptualization:** Ning Xuan.

**Data curation:** Yuehang Fan.

**Formal analysis:** Yuji Jiang.

**Methodology:** Yuehang Fan, Xiangyu Meng.

**Software:** Yuehang Fan.

**Supervision:** Xiangyu Meng, Yuji Jiang.

**Writing – original draft:** Tian Zou, Ning Xuan.

**Writing – review & editing:** Tian Zou, Yuehang Fan, Xiangyu Meng, Yuji Jiang.










